# Tuina therapy for chronic nonspecific low back pain: a randomized clinical trial protocol

**DOI:** 10.3389/fmed.2026.1839845

**Published:** 2026-05-07

**Authors:** Siwei Zhou, Changzheng Jiang, Qiangwen Xie, Bang Zhan, Lin Li, Shuijin Chen, Lechun Chen, Zhigang Lin

**Affiliations:** 1 Fujian University of Traditional Chinese Medicine, Fuzhou, Fujian, China; 2 Massage Department, Rehabilitation Hospital Affiliated to Fujian University of Traditional Chinese Medicine, Fuzhou, Fujian, China; 3 Fujian Key Laboratory of Rehabilitation Technology, Fuzhou, Fujian, China

**Keywords:** CNLBP, Loxoprofen Sodium patches, muscle, randomized controlled trial, Tuina

## Abstract

**Background:**

Chronic Non-Specific Low Back Pain (CNLBP) is a prevalent musculoskeletal condition. Tuina therapy and non-steroidal anti-inflammatory drugs (NSAIDs) are two commonly used treatments for CNLBP, both of which have demonstrated some clinical efficacy. However, the effectiveness, safety, and potential opioid-sparing effects of Tuina, a classic complementary and alternative therapy, have yet to be rigorously validated through clinical research.

**Objective:**

This study aims to conduct a randomized controlled superiority trial with an open-label design, blinded assessment, and parallel groups to evaluate the effectiveness, safety, and complementary role of Tuina in the treatment of CNLBP.

**Study design and methods:**

This is a single-center, randomized controlled, assessor-blind clinical trial. A total of 80 participants aged 18–60 years meeting the diagnostic criteria for CNLBP will be recruited and randomly assigned to either the experimental group (Tuina combined with Loxoprofen Sodium patches) or the control group (Loxoprofen Sodium patches). The Tuina intervention will be administered three times weekly for four consecutive weeks; Loxoprofen Sodium patches will be used as needed. The primary outcome measure is the Visual Analogue Scale (VAS) for pain after 4 weeks of treatment. Secondary outcomes include the Oswestry Disability Index (ODI), 36-Item Short Form Health Survey (SF-36), Beck Depression Inventory-Second Edition (BDI-II), Beck Anxiety Inventory (BAI), Surface Electromyography (sEMG) and musculoskeletal ultrasound (MSK US) parameters, medication usage, and 12-week follow-up data. Statistical analysis will follow the intention-to-treat (ITT) principle, using SPSS 26.0 for between-group comparisons and repeated measures analysis.

**Discussion:**

Through a rigorous randomized controlled design incorporating both physiological and morphological objective indicators, this study aims to provide high-level evidence regarding the analgesic efficacy, safety, and potential for drug dose reduction of Tuina in treating CNLBP. It also seeks to offer clinical evidence and practical references for the application of Tuina in the rehabilitation of musculoskeletal disorders.

**Clinical trial registration:**

https://itmctr.ccebtcm.org.cn/mgt/project/user/user-project-view/4621255a-332d-4ba4-b09b-e9412ba06d0b, Identifier (ITMCTR2026000745).

## Introduction

Chronic non-specific low back pain is typically defined as pain, muscle tension, or stiffness lasting for more than 12 weeks, located between the costal margin and the inferior gluteal folds, which cannot be attributed to a specific pathological cause, such as nerve root compression, fracture, infection, tumor, inflammatory disease, etc. Through imaging or physical examination (1). Epidemiological data indicate that chronic non-specific low back pain is the leading cause of healthy life years lost worldwide (2, 3). Although the age-standardized prevalence rate has remained stable or even slightly decreased over the past three decades, the total number of affected individuals has significantly increased due to population growth and ageing demographics (4). This condition is most common in middle-aged populations, with a peak incidence between 50 and 54 years, and the risk of developing CNLBP is generally higher in women than in men. In older adults, the risk factors become more complex, encompassing demographic characteristics, psychological status, lifestyle, and occupational exposures (5). CNLBP leads to widespread functional disability and reduced health-related quality of life. It is one of the leading causes of work absenteeism and activity limitation globally, thereby significantly increasing individual disease burden and socio-economic costs (6–8).

A key pathological feature of CNLBP is the imbalance in the function and morphology of the lumbar muscles. Clinical imaging evidence shows that patients with chronic low back pain often experience a significant decline in lumbar muscle mass, characterized by atrophy of the multifidus muscle, reduced cross-sectional area, and increased intra-muscular fat infiltration (9). Simultaneously, the superficial erector spinae muscles also exhibit characteristic imaging changes. Some scholars believe that the erector spinae in CNLBP patients typically show significant fatty infiltration, with elevated fat fraction levels correlating with the degree of pain and dysfunction (10). In animal models, chronic low back pain associated with intervertebral disc degeneration can induce structural changes in the paraspinal muscles, particularly the multifidus. These changes include alterations in muscle fibre types, for example, a reduction in type I fibres and an increase in type IIX fibres and structural degeneration such as muscle fibrosis (11). It should be noted, however, that the diagnostic criteria for CNLBP explicitly exclude specific disc pathologies (e.g., disc herniation with nerve root compression) as a primary cause of pain. Therefore, findings from disc degeneration-based animal models may not be directly generalizable to the CNLBP population. Nonetheless, these observations provide valuable insights into potential paraspinal muscle adaptations that may occur in chronic low back pain conditions.

Tuina, a traditional Chinese manual therapy, offers unique advantages in the clinical management of musculoskeletal disorders. Multiple clinical studies have shown that Tuina can effectively alleviate pain and improve physical function in patients with chronic low back pain (12). Its mechanisms may involve inhibiting skeletal muscle fibrosis, modulating various signaling pathways and cytokine expression, and promoting muscle tissue repair (13). Furthermore, animal experiments have indicated that in rat models of nerve injury, Tuina can effectively slow down skeletal muscle atrophy resulting from denervation (14).

CNLBP is characterized by inhibition and atrophy of the deep stabilizing muscles, such as the multifidus, along with compensatory tension in the superficial mobilizing muscles, such as the erector spinae, which leads to persistent pain and reduced spinal stability. Tuina techniques, through deep tissue manipulation, can help alleviate myofascial adhesions, reduce abnormal muscle tone, and restore tissue gliding. Specific stimulation directed at deep muscles may facilitate the reactivation of their neuromuscular control (15, 16). Therefore, Tuina provides a foundational approach for rehabilitating muscle function in patients with CNLBP.

Currently, high-quality clinical trials on Tuina for CNLBP are still lacking. Previous systematic reviews and meta-analyses have pointed out limitations in existing studies, such as inadequate safety monitoring, unclear outcome measures, loss to follow-up, small sample sizes, and limited evidence quality (17). Although Tuina shows promise as an independent or complementary treatment, high-quality systematic evidence is still scarce regarding whether its combination with conventional pharmacological treatments such as NSAID patches, which provide foundational analgesia that may reduce movement-evoked pain, thereby creating a favorable condition for Tuina to promote muscle function recovery (18, 19). To address this gap, the present study aims to systematically evaluate the comprehensive efficacy of Tuina combined with Loxoprofen Sodium patches for treating CNLBP using an assessment system integrating subjective and objective measures. The focus is on exploring the analgesic value of Tuina therapy on patients with CNLBP and its impact on lumbar muscle function remodeling, with the goal of providing more substantial evidence for clinical practice.

## Methods

### Study design

**Figure 1:**
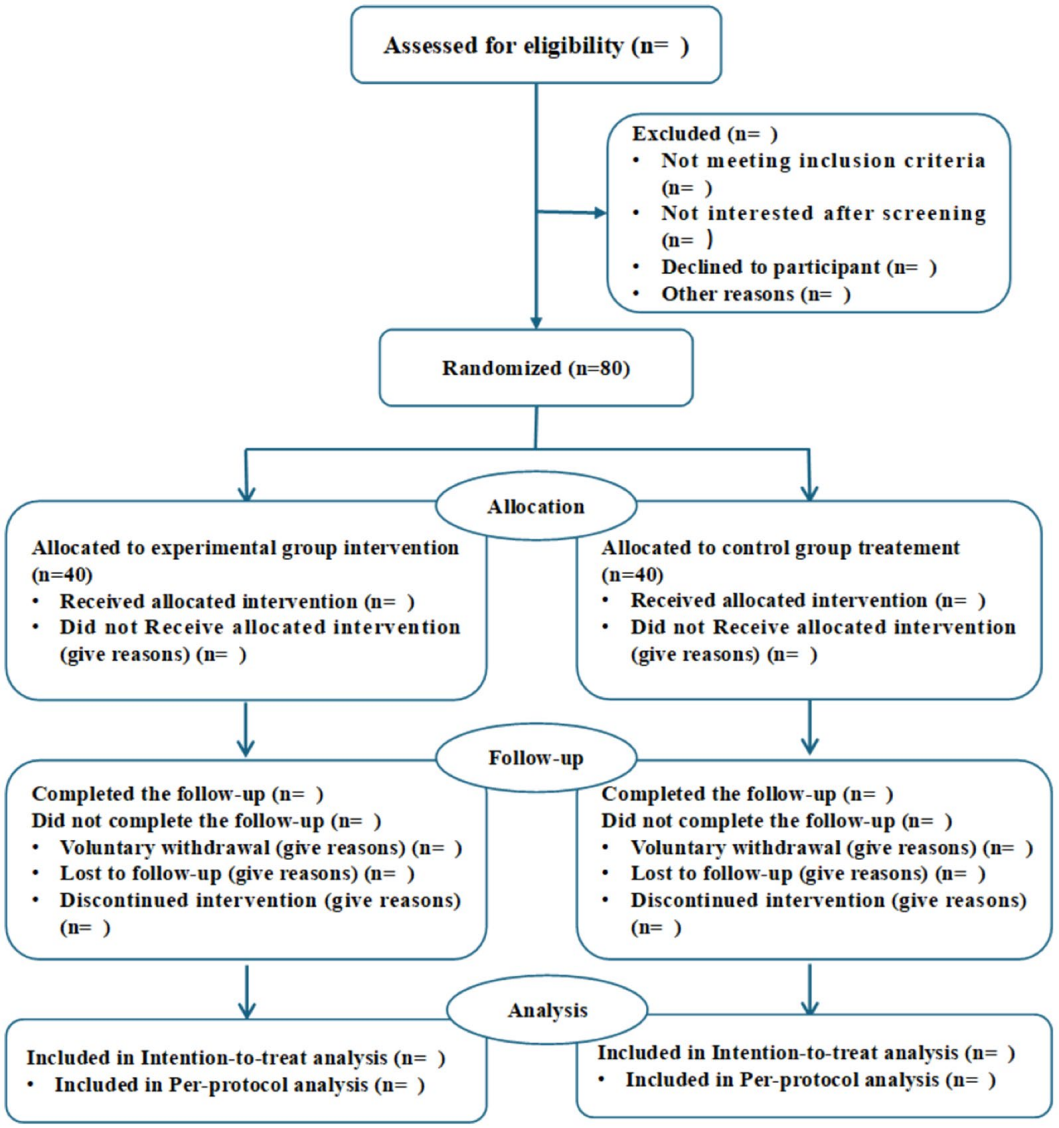
Study flow chart.

This is a two-arm randomized controlled superiority trial to be conducted at a single center in China. A total of 80 patients with CNLBP will be randomly assigned in a 1:1 ratio to either the experimental group (Tuina combined with Loxoprofen Sodium patches) or the control group (Loxoprofen Sodium patches alone). The trial duration is 16 weeks, comprising a 4-week treatment period and a 12-week follow-up period. This study protocol has been approved by the Ethics Committee of the Rehabilitation Hospital Affiliated to Fujian University of Traditional Chinese Medicine (Approval Number: 2025KY-031-02). This trial follows the CONSORT (Consolidated Standards of Reporting Trials) guidelines (see Figure 1).

### Study site

Recruitment was conducted concurrently through the Outpatient Department of the Rehabilitation Hospital affiliated with Fujian University of Traditional Chinese Medicine and its official WeChat public account. All interventions and follow-ups were performed at the Rehabilitation Hospital affiliated with Fujian University of Traditional Chinese Medicine. Following randomization, participants were assigned to their respective groups and underwent the intervention and follow-up procedures in individual, closed-door clinical examination rooms to ensure patient privacy and minimize environmental distractions.

### Participants

#### Diagnostic criteria

Diagnosis was based on the 2020 Evidence-Based Clinical Guidelines for Non-specific Low Back Pain developed by the North American Spine Society (NASS) (20). The criteria included: (1) a disease course exceeding 3 months, with recurrent episodes or chronic persistence; (2) no identifiable underlying cause, with no evidence of nerve root involvement or spinal stenosis; (3) pain and discomfort localized to the area between the costal margins inferiorly, the gluteal folds superiorly, and the bilateral mid-axillary lines laterally, with or without referred pain to the lower limbs; (4) physical examination revealing localized or diffuse tenderness or percussion pain over the lumbosacral region; (5) Specific severe spinal lesions (such as fractures, tumors, infections, cauda equina syndrome, progressive neurological deficits) are excluded, however, routine age-related degenerative changes (such as intervertebral disc degeneration, disc herniation, facet joint osteoarthritis, Schmel’s knot, etc.) are not excluded; (6) neurological examination typically revealing no sensory, motor, or tendon reflex deficits.

#### Inclusion criteria

Eligible participants meeting all of the following criteria were enrolled: (1) met the diagnostic criteria outlined above; (2) aged between 18 and 60 years; (3) had a VAS score ≥ 40 mm within the preceding 7 days; (4) had not received any Traditional Chinese Medicine or Western medical treatment related to low back pain in the preceding 4 weeks; (5) provided signed informed consent.

#### Exclusion criteria

Participants meeting any of the following criteria were excluded: (1) low back pain secondary to diagnosed internal medical conditions or previous lumbar spine surgery, confirmed by relevant laboratory and imaging examinations; (2) patients with severe osteoporosis, spinal fractures, inflammatory spondyloarthritis (such as ankylosing spondylitis), spondylolisthesis, lumbar spinal stenosis, and lumbar disc herniation; (3) presence of severe cardiac, pulmonary, hepatic, or renal insufficiency or significant impairment, or coagulation disorders, or history of active peptic ulcer, severe gastrointestinal bleeding, active inflammatory bowel disease, and aspirin-induced asthma; (4) inability to comply with the study protocol due to severe psychiatric illness or cognitive impairment; (5) known allergy to non-steroidal anti-inflammatory drugs (NSAIDs), alcohol, or electrode pads, or patients taking medications that severely interact with NSAIDs like Loxoprofen, such as anticoagulants and antiplatelets, lithium or methotrexate, and SSRIs; (6) women who were pregnant or breastfeeding.

### Allocation

Eighty patients with CNLBP were simply randomly assigned to groups using a random number generator from SPSS 26.0 statistical software. Random allocation cards were placed in opaque envelopes, which are sequentially numbered on the outside, sealed with tape, and the contents cannot be read under strong light. The generated random number table was securely stored by a designated individual not involved in clinical interventions or outcome assessments. During the clinical trial, envelopes were sequentially opened based on patients’ presentation order, and corresponding treatments were administered according to the assigned group. The sequentially numbered envelope for a participant is only opened after baseline assessments and informed consent have been fully completed.

### Blinding

Due to the specific nature of the manipulative intervention, blinding of participants and practitioners was not feasible. Therefore, this study implements blinding only for the objective outcome assessments and statistical analysis. Specifically, the assessors of sEMG and MSK US, as well as the data analysts, are unaware of group assignments and treatment details. However, patient-reported outcome measures, including VAS, ODI, SF-36, BDI-II, and BAI, are self-reported by participants who are aware of their treatment allocation; thus, these subjective outcomes remain at risk of detection bias. A three-separation principle—separating clinical intervention, objective outcome evaluation, and data analysis—was implemented to minimize bias where feasible. To protect the blind, objective assessors are instructed to avoid discussing treatment-related details with participants, and participants are reminded not to reveal their group assignment during assessments.

### Interventions

Researchers conducting the intervention will record participants’ attendance frequency in the intervention diary but will not collect any additional data for the experiment. If a participant misses an intervention session, we will promptly contact them to determine the reason and encourage them to complete the scheduled intervention. This study adopts an explanatory trial design to evaluate the specific therapeutic effects of Tuina combined with Loxoprofen Sodium patches under controlled conditions. Throughout the research period, participants are prohibited from seeking other conventional treatments for CNLBP after consulting with the researchers. Researchers will document participants who receive such additional treatments and withdraw them from the study intervention. However, in accordance with the ITT principle, these participants will be retained in the trial for follow-up and included in the primary data analysis.

#### Experimental group

**Figure 2:**
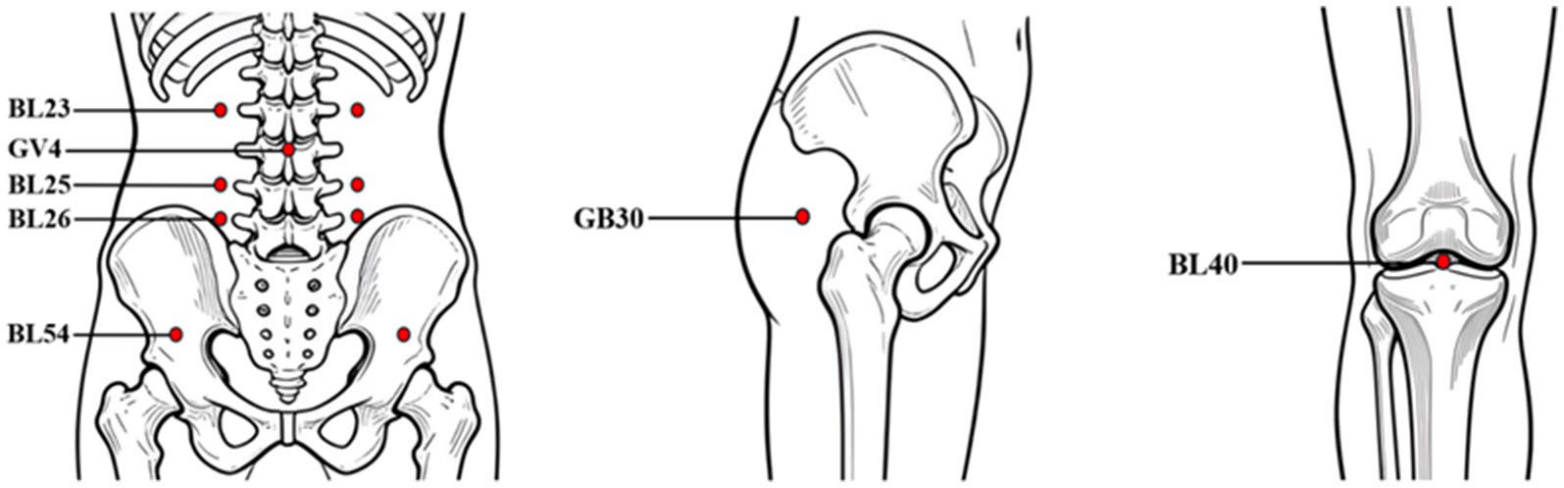
Location of acupoints.

The experimental group received Tuina combined with the Loxoprofen Sodium patch. The Tuina intervention will be performed by a physician with over 10 years of experience in Tuina treatment from the Department of Tuina, the Affiliated Rehabilitation Hospital of Fujian University of Traditional Chinese Medicine. The patient is first guided to assume a prone position. The Tuina therapist sequentially applies the rolling manipulation and palmar pressing-kneading manipulation to the Bladder Meridian on both sides of the lower back and the posterolateral aspect of the affected lower limb. Each region is treated for 5 min, totaling 10 min, to alleviate muscle tension. Maintaining the same position, finger-pressing manipulation is applied to the acupoints of Ashi, BL23 (Shenshu), GV4 (Mingmen), BL25 (Dachangshu), BL26 (Guanyuanshu), BL54 (Zhibian), GB30 (Huantiao), BL40 (Weizhong): (see Figure 2 and Table 1), with force generated from the forearm and fingers. The intensity is adjusted to the patient’s comfort level, and rhythmic pressing and kneading are performed for 5 min. The patient is then guided to a lateral recumbent position. The Tuina therapist first applies thumb-plucking manipulation to the bilateral erector spine muscles for 3 to 5 repetitions, followed by lumbar oblique-pulling manipulation once on each side, until the audible sound of bubbles (“click” sound) in the joint cavity can be heard at the end of the operation. Subsequently, the patient returns to the prone position, and the bilateral sacrospinal muscles are patted, followed by tapping on the lumbosacral region to relieve spasm, alleviate pain, and relax the sinews and unblock the meridians. The intervention is administered three times weekly, with each session lasting 30 min, for a continuous treatment duration of 4 weeks.

**Table 1:** Location of acupoints.

Acupoints	Location
BL23 (Shenshu)	On the back, at the same level as the lower border of the spinous process of the second lumbar vertebra (L2), 1.5 cun lateral to the posterior midline.
GV4 (Mingmen)	On the posterior midline, in the depression inferior to the spinous process of the second lumbar vertebra (L2).
BL25 (Dachangshu)	On the back, at the same level as the lower border of the spinous process of the fourth lumbar vertebra (L4), 1.5 cun lateral to the posterior midline.
BL26 (Guanyuanshu)	On the back, at the same level as the lower border of the spinous process of the fifth lumbar vertebra (L5), 1.5 cun lateral to the posterior midline.
BL54 (Zhibian)	On the buttock, lateral to the sacral hiatus, at the level of the fourth posterior sacral foramen, 3 cun lateral to the posterior midline.
GB30 (Huantiao)	On the lateral side of the buttock, at the junction of the lateral one-third and medial two-thirds of the distance between the prominent point of the greater trochanter and the sacral hiatus.
BL40 (Weizhong)	On the posterior aspect of the knee, at the midpoint of the popliteal crease.

**Figure 3:**
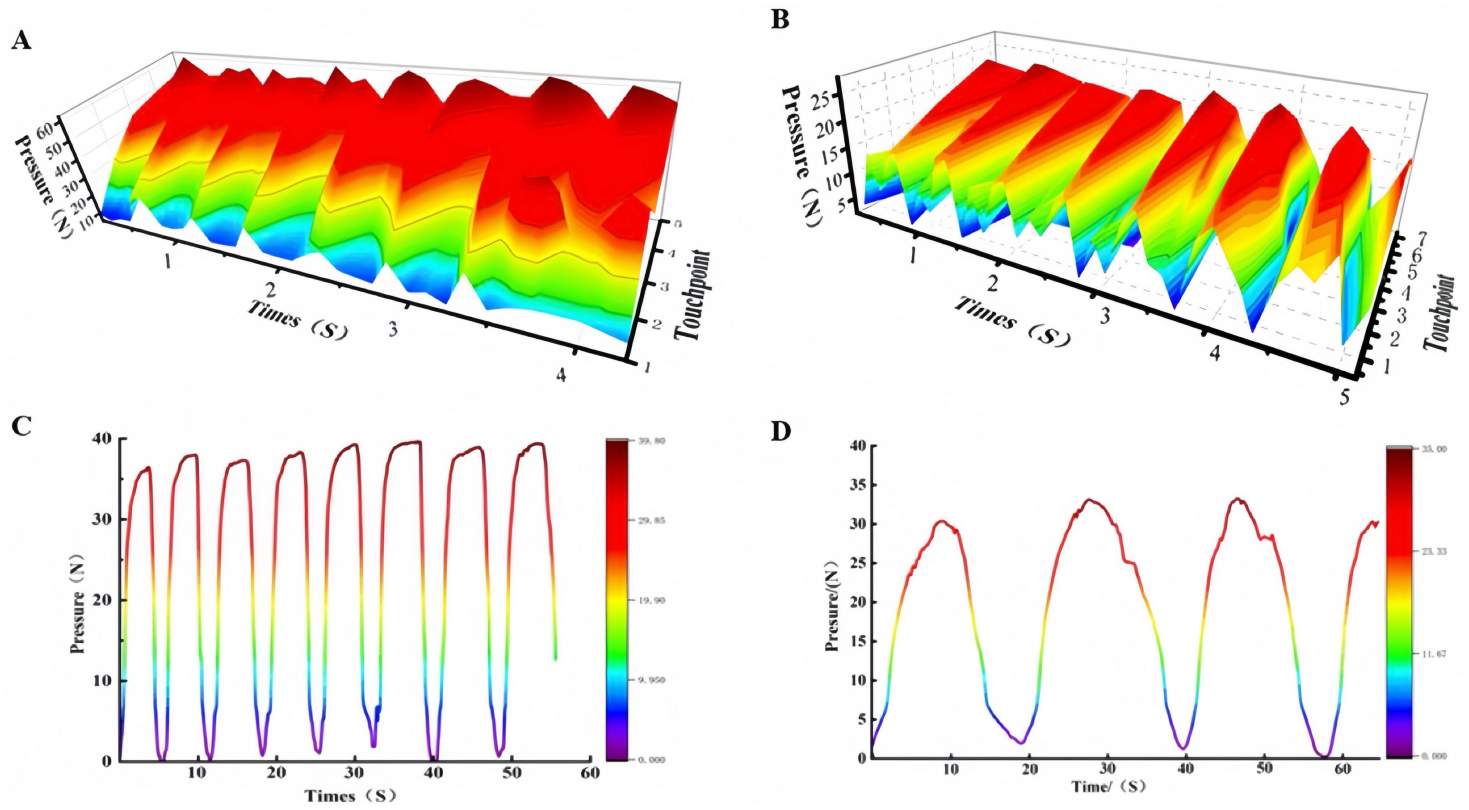
Pressure graph of Tuina technique operation parameters. **(A)** Pressure graph of the rolling manipulation; **(B)** pressure graph of the pressing-kneading manipulation; **(C)** pressure graph of the finger-pressing manipulation; **(D)** pressure graph of the thumb-plucking manipulation.

Before starting the treatment intervention, the Tuina therapist used smart gloves for a month of manual practice to standardize each Tuina technique and parameter level. Before treating each patient, the massage therapist will wear gloves on the operating table for 5 min to standardize the Tuina technique again. After completing the exercise, the Tuina therapist should rest for 5 min before starting the Tuina treatment. The actual force applied to the patient relies on the therapist’s conditioned muscle memory. Referring to relevant literature, the specific operating parameters are as follows (see Figure 3):

(1) *Rolling manipulation*: the practitioner assumes a T-step stance with shoulders relaxed, elbows dropped, arms held vertically, and palms erect. The dorsum of the little finger’s metacarpophalangeal joint is fixed on the treatment area. Active flexion and extension of the elbow joint drive the forearm and wrist in a combined motion of external rotation with flexion and internal rotation with extension. This causes the hypothenar region and the ulnar half of the hand dorsum to roll back and forth over the treatment area. The manipulation is performed at a frequency of 120 times per minute with a force of 30 ~ 50 N.
(2) *Palmar pressing-kneading manipulation*: the practitioner fixes the thenar eminence on the treatment area. Active flexion and extension of the elbow joint drive the forearm, which in turn drives the wrist, and the wrist drives the thenar eminence. The thenar eminence should remain in close contact with the skin surface, driving the subcutaneous tissue without any dragging, friction, or slipping over the skin. The operating frequency is generally 120–160 times per minute with a force of 15 ~ 25 N.
(3) *Finger-pressing manipulation*: the practitioner applies pressure to the treatment area using the tip of the thumb, the tip of the middle finger, or the tips of both thumbs (one on top of the other). Pressure is applied downward slowly, progressing from superficial to deep and from light to heavy. Achieve the therapeutic’s conditioned muscle memory strength value, the pressure is maintained for 3 s and then released slowly. The manipulation is performed at a frequency of 20 times per minute with a force of 10 ~ 20 N.
(4) *Thumb-plucking manipulation*: the practitioner applies firm pressure with the thumb, or with the index and middle fingers, or with the index, middle, and ring fingers, palpate along one side of a palpable cord-like structure, such as a taut muscle band or tendon, within the recipient’s body. Perpendicular to the structure’s direction, the practitioner then plucks it unidirectionally or back and forth. During the manipulation, the force should be applied gradually from light to heavy, and then reduced from heavy to light, in a wave-like pattern of rising and falling. If the force of a single thumb is insufficient, both thumbs can be used together in an overlapping manner. The manipulation is performed at a frequency of 20 times per minute with a force of 30 ~ 40 N.

During the 4-week treatment period, Loxoprofen Sodium Patch [Specification: 50 mg/patch, Manufacturer: Daiichi Sankyo (China) Investment Co., Ltd., China Drug Approval Number: H20040130] was applied as needed (pro re nata) once daily to the painful area of the lower back on days when pain intensity became bothersome or exceeded a tolerable baseline, avoiding use within 2 h after massage treatment, and avoiding application to any area with bruising or abrasions. It was used upon the onset of pain and discontinued upon pain relief (pro re nata, PRN). Participants were also required to complete a medication record form.

#### Control group

During the 4-week treatment period, the control group received the Loxoprofen Sodium Patch [Specification: 50 mg/patch, Manufacturer: Daiichi Sankyo (China) Investment Co., Ltd., China Drug Approval Number: H20040130] was applied as needed (pro re nata) once daily to the painful area of the lower back on days when pain intensity became bothersome or exceeded a tolerable baseline, and avoiding application to any area with bruising or abrasions. It was applied to the painful area of the lower back once daily upon the onset of pain and discontinued upon pain relief. Participants were also required to complete a medication record form.

#### Primary outcome

Primary Outcome: The primary outcome measure of this trial is the change in pain intensity from baseline to the end of the 4-week treatment period, assessed via a VAS (21). The VAS is a sensitive, continuous, and reliable instrument for quantifying subjective pain perception. A standard 100-mm VAS will be employed, anchored by the verbal descriptors “0 mm = no pain” at the left end and “100 mm = the worst pain imaginable” at the right end. To account for the fluctuating nature of chronic low back pain, patients will be instructed to use the VAS to rate their average pain intensity over the past week. A higher measurement (in millimeters) indicates greater pain severity.

#### Secondary outcomes

Secondary outcome measures included ODI questionnaire, SF-36, BDI-II, BAI, sEMG, MSK US, and medication usage.

**Figure 4:**
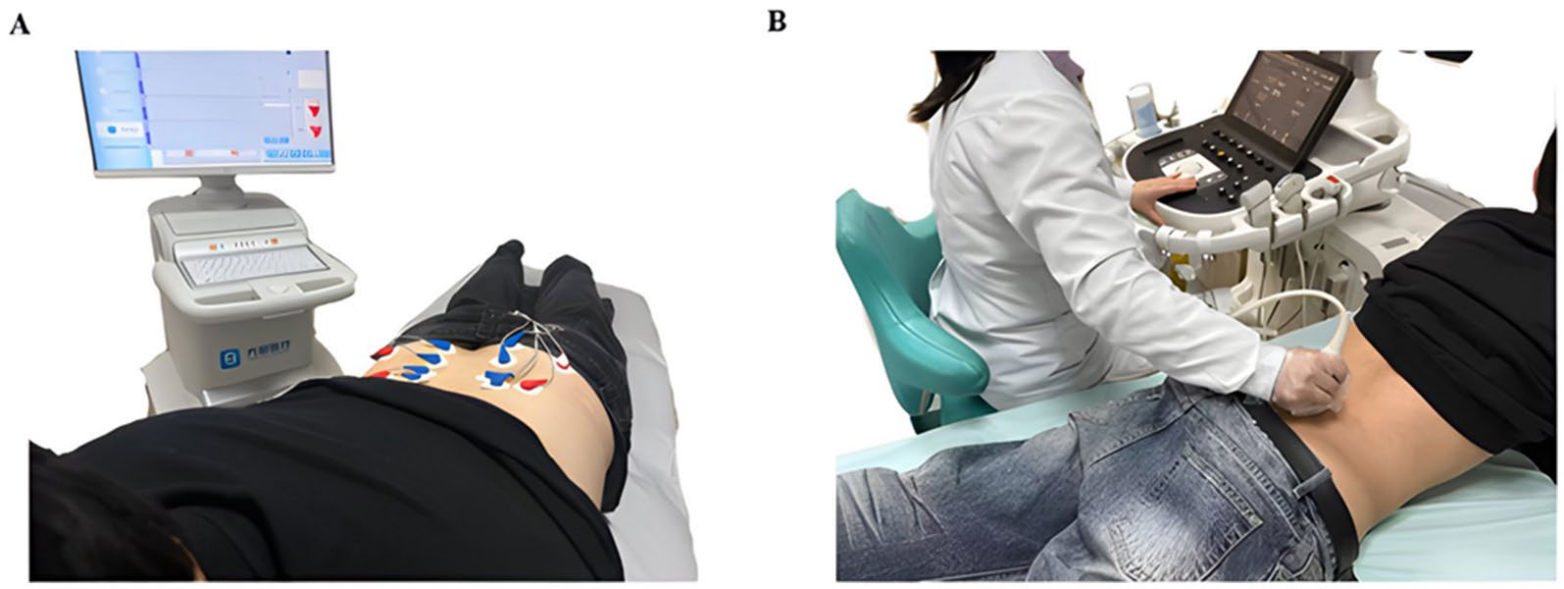
Examples of sEMG and MSK US inspection operations. **(A)** Examples of sEMG inspection operations. **(B)** Examples of MSK US inspection operations.

1. ODI Questionnaire (22, 23): This questionnaire is considered a standard measure for all LBP. It evaluates various aspects, including the intensity of low back and leg pain, daily activities, and social life. The ODI 2.0 version will be used in this trial, as it is the most commonly employed version in low back pain research and has well-established psychometric properties. The ODI score ranges from 0 to 100%. The final score is inversely related to the level of lumbar function; a higher index indicates more severe dysfunction, whereas a lower index suggests milder dysfunction.
2. SF-36 (24): The full version of the SF-36 is a widely used generic health assessment tool. Its physical health component comprises four domains: Physical Functioning (assessing capacity for daily activities), Role-Physical (reflecting the impact of health problems on work or daily life), Bodily Pain (quantifying pain intensity and its interference), and General Health Perceptions (evaluating the individual’s overall view of their health). The standard (4-week recall) version will be administered, as it is appropriate for chronic conditions where stable health status over the past month is of interest. Scores for each item are transformed using a standardized algorithm to a 0–100 scale, with higher scores indicating better physical health status.
3. BDI-II (1): This is a self-report inventory used to assess the severity of depressive symptoms over a 2-week recall period. It contains 21 items covering emotional, cognitive, and somatic symptoms, such as hopelessness, self-blame, and sleep disturbances. Each item is rated on a 0–3 scale, yielding a total score range of 0–63. Higher scores reflect more severe depressive symptoms.
4. BAI (25): This is a self-report tool for assessing the severity of anxiety symptoms. It comprises 21 items focusing on physiological and emotional symptoms, for example, palpitations, trembling, feelings of nervousness. Each item is scored from 0 to 3, with total scores ranging from 0 to 63. Higher scores indicate more severe anxiety levels.
5. sEMG: Measurements included the root mean square (RMS) values of the bilateral erector spinae and bilateral multifidus muscles; amplitude-frequency changes; negative slope changes in median frequency; and bilateral differences in RMS values or activation timing. sEMG electrodes were placed over the bilateral erector spinae (approximately 2–3 cm lateral to the L1 and L3 vertebral levels) and the bilateral multifidus (at the L5 level). sEMG signals were recorded continuously and synchronously throughout the execution of a full prone trunk extension held test by the participants. The acquired sEMG RMS values quantified the level of muscle activation. Analyzed time-domain indices primarily included the mean amplitude of the electromyographic signal, reflecting the trend of muscle activation intensity throughout the test. Frequency-domain indices, such as the median frequency, were used to quantify the rate of muscle fatigue during sustained contraction. Bilateral differences in RMS values or activation timing were analyzed to assess disordered muscle activation sequences or compensatory patterns in patients with low back pain (see Figure 2).
6. MSK US: Measurements included muscle thickness and cross-sectional area of the bilateral erector spinae and multifidus; shear wave elastography; and muscle echogenicity and homogeneity. MSK US was used to assess the bilateral multifidus and bilateral erector spinae. Images were acquired at three times: before, during, and after the participant performed the prone trunk extension hold test. In the resting state, participants lay prone in a completely relaxed position. Transverse images at the L4-L5 level were used to measure the thickness and cross-sectional area of the multifidus muscle and the bilateral erector spinae muscle. Shear wave elastography was employed to obtain elasticity modulus values or shear wave speeds to quantify muscle stiffness. During the maintenance phase of the test movement at 30 and 60 s, a brief pause allowed for rapid acquisition of images depicting muscle morphology and elasticity during isometric contraction. Muscle echogenicity and homogeneity will be objectively quantified using grayscale histogram analysis. Immediately following the test, resting state parameters were measured again to assess the impact of acute fatigue on muscle status. Probe positioning and skin markings were kept consistent for all measurements to ensure data comparability (see Figure 4).
7. Medication usage: The weekly frequency and usage dosage of medication during the study period were recorded for both the control and treatment groups. These data will be quantitatively analyzed to evaluate the medication use of Tuina intervention.

The VAS is assessed prior to the first treatment and after each subsequent treatment session. Medication usage was evaluated before the initial treatment and after the second, third, and fourth weeks of treatment. The ODI, SF-36, BDI-II, BAI, sEMG, and MSK US assessments were performed for patients with chronic nonspecific low back pain prior to the first treatment and upon completion of the treatment phase. Additionally, follow-up evaluations were conducted at 4, 8, and 12 weeks after the conclusion of the treatment intervention, primarily collecting data on patients’ pain VAS, ODI, and medication usage during clinical visits (see Table 2).

**Table 2:** The schedule of enrollment, interventions, and assessment.

Visit item	Enrollment	Allocation	Treatment period					Follow-up period		
Time point	−3–0 day	0 day	1 day	1 week	2 week	3 week	4 week	8 week	12 week	16 Week
Register	✓									
Sign the informed consent form		✓								
General information		✓								
Inclusion and exclusion criteria		✓								
Randomization		✓								
Basic information, current medical history, past medical history		✓								
Intervation										
Tuina combined with Loxoprofen Sodium patches			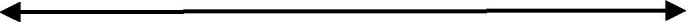							
Loxoprofen Sodium patches			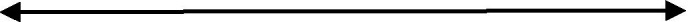							
Assessments										
VAS		✓	✓	✓	✓	✓	√	√	√	√
ODI		✓					√	√	√	√
SF-36		✓					√			
sEMG		✓					√			
BDI-II		✓					√			
BAI		✓					√			
MSK-US		✓					√			
Medication usage		✓		✓	✓	✓	√	√	√	√
Safety evaluation										
Adverse events			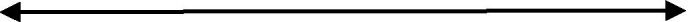							

### Adverse events

In this study, the monitoring, assessment, and management of adverse events related to Tuina followed a comprehensive standard operating procedure. Initially, any discomfort reported during the intervention period was promptly evaluated and judged by at least two clinical experts with senior professional titles from our research group. According to previous literature and clinical consensus, common adverse events associated with Tuina manipulation primarily include worsening local pain or discomfort, skin redness or ecchymosis, transient muscle soreness, and, in rare instances, temporary dizziness or fatigue; the adverse reactions associated with Loxoprofen Sodium patches, including gastrointestinal discomfort, contact dermatitis, and erythema at the application site. Upon confirmation of any adverse event, a detailed record was made, and the event was reported to the study’s ethics committee within 24 h.

Management was implemented based on the severity of the event using a graded approach: For mild local discomfort, participants could continue in the study under close observation. For moderate pain exacerbation or persistent dizziness, the current treatment cycle was immediately suspended, and symptomatic treatment was provided until complete symptom resolution and safety were confirmed through evaluation. In the event of a serious adverse event, the research intervention for that subject was immediately and permanently terminated, emergency procedures were initiated, and reports were submitted to the relevant authorities within the mandated timeframe. Furthermore, if prohibited concomitant treatments, such as the systemic use of other analgesic medications or receiving other physical therapies, were used to manage an adverse event, or if the event led to the subject being unable to complete follow-up for primary outcome measures, including pain scores, functional disability indices, and surface electromyography assessments, the data from lost-to-follow-up participants will be supplemented using multiple imputation and included in the final efficacy analysis. Their safety data were retained and reported.

### Patient and public involvement

In this study protocol, participants and the general public will not be involved in all stages of the study. The role of participants will be confined to those aspects directly relevant to the core objectives of the research, and they will not be engaged in the recruitment, implementation, or reporting phases of the study. Their search findings will be disseminated to participants and the public through educational lectures, brochures, or pamphlets. Moreover, the results will be published in open-access, peer-reviewed journals.

### Data management

Each enrolled subject was required to have a completed Case Report Form (CRF). Dedicated assessment personnel entered data into the CRF completely, truthfully, and accurately. After verification and signature by the clinical research monitor, the CRF was promptly transferred to the data administrator, who then accurately entered the CRF data into the database. This database automatically validates the input data and flags any inconsistent or questionable data, after which the data administrator sends a query request to the researchers, and upon the researchers’ approval, these data are checked and corrected. All data were entered independently by two individuals in duplicate. The software automatically verified entries and flagged any inconsistent or questionable data, after which the data administrator sends a query request to the researchers, and upon the researchers’ approval, these data are checked and corrected. A random 10% of the CRFs were subjected to manual verification. After all data were confirmed to be accurate, the database was locked by the principal investigator, statistician, and the person responsible for blinding. No further modifications were permitted to the locked data files. Regarding data preservation, investigators were required to retained for a minimum of 5 years after the completion of the trial all study materials, including all original source data such as imaging discs, data files, CRFs, and informed consent forms.

### Sample size

Based on the study design, the VAS score after the 4-week treatment was designated as the primary outcome measure. According to the data from preliminary pilot studies, after 4 weeks of intervention, the VAS score of 26.2 ± 13.7 mm for the experimental group and 36.4 ± 14.8 mm for the control group. Using G*Power 3.1^1^ software to estimate the sample size, a two-independent-sample *t*-test was selected for estimation. Setting *α* = 0.05, *β* = 0.2, and using a two-sided statistical test, the calculated effect size d was 0.804, based on which it was estimated that 32 participants are needed per group. Considering a potential dropout rate of 20%, the final sample size was determined to be 40 participants per group, totaling 80 participants.

### Statistical analysis

Statistical analysis will follow the ITT principle, including all randomly assigned participants. Data from all lost-to-follow-up participants will be completed using multiple imputation, and statistical analysis will be conducted using SPSS 26.0 software. All continuous variables will be first tested for normality. Normally distributed data were presented as mean ± SD, and t-tests were performed for statistical analysis; while non-normally distributed data will be presented as M (P25, P75), and statistical analysis was performed using the Mann–Whitney U test. Categorical variables will be expressed as frequencies or percentages, and statistical analysis was performed using the chi-square test. The longitudinal analysis will be performed using a linear mixed model with baseline value as covariate. A two-sided *p*-value of <0.05 will be considered statistically significant. To address the problem of multiple Type I errors, the Bonferroni correction method will be used to adjust the significance level.

For safety analysis, the number of cases with abnormal changes in laboratory indicators will be described, and the incidence, presentation, severity, and correlation with the intervention will be reported in detail for all adverse events.

### Trial status

The trial is currently in the participant recruitment phase. Following trial registration, the first participant was enrolled on October 1, 2025. Recruitment is expected to be completed by June 30, 2027. As of the submission date of this protocol manuscript, a total of 9 participants have been successfully enrolled. We hereby declare that no interim data analysis has been performed, the database remains open and unlocked, and all researchers and statisticians are completely blinded to the accumulated outcome data at the time of submission.

## Discussion

CNLBP represents a persistent individual burden and is a significant contributor to socioeconomic costs. Tuina, one of the most commonly utilized complementary therapies, and nonsteroidal anti-inflammatory drugs (NSAIDs), a core intervention recommended in clinical guidelines, are both treatment modalities for CNLBP. While non-pharmacological treatments are often preferred, and numerous studies have confirmed the benefits of NSAIDs (26), research directly comparing these two approaches to guide individualized treatment for specific patient populations remains scarce.

In clinical practice, many patients with CNLBP seek physical therapies such as Tuina, either alongside or instead of pharmacological treatment. Existing clinical guidelines frequently position medication as a foundational approach, while Tuina is also a widely applied intervention. Numerous studies have separately confirmed the efficacy of medication and Tuina. However, there is a notable lack of research rigorously comparing combination therapy (Tuina plus medication) against medication alone. Such comparative studies hold significant practical value for guiding individualized clinical treatment choices. This study introduces the use of specialized Tuina gloves to standardize the manipulation technique, directly addressing the critical challenge of achieving uniformity and reproducibility in clinical Tuina practice.

Given that the core clinical issues in CNLBP are pain and functional limitation, and to clearly elucidate the differences in efficacy between the two treatment protocols, this study designates pain intensity as the sole primary outcome (VAS), and muscle function represents an objective secondary outcome. This approach allows for a comprehensive evaluation encompassing both the patient’s subjective experience and the objective improvement in physical function. Furthermore, the study will delve into the specific changes in muscle function following Tuina intervention, aiming to provide evidence-based insights into the potential mechanisms underlying its therapeutic effect in CNLBP.

This study has certain limitations. First, due to the open-label nature of Tuina, blinding of patients and therapists is not feasible. Additionally, this single-center trial is conducted at a TCM-affiliated hospital and lacks an attention-matched control group, where enrolled patients likely have a high baseline preference for Tuina, which may amplify expectation bias and placebo effects in the experimental group. Therefore, our findings may not be fully generalizable to populations or settings with lower familiarity with TCM manual therapy. Although outcome assessors and data analysts are blinded to minimize bias in objective biomechanical data, this does not reduce measurement bias for the primary outcome (VAS). Second, constrained by the follow-up duration, a 12-week observation period may be insufficient to fully capture the long-term effects of the treatment regimen for a chronic condition such as CNLBP. Therefore, further studies with longer follow-up are necessary to validate the long-term differences between medication alone and Tuina combined with medication. Third, the two-arm design lacks a Tuina-alone control and a step-up phase. Thus, any observed benefit of combination therapy may arise from Tuina alone or non-specific effects rather than true synergy. Future trials should address these limitations using three-arm, run-in, and sham-controlled designs. Fourth, the inclusion age is capped at 60 years to reduce confounding from age-related degenerative pathologies. Consequently, our findings may not be generalizable to geriatric patients.

This study aims to clarify the clinical effectiveness of combining Tuina with medication for treating CNLBP. The findings are expected to provide a scientific reference to inform clinical decision-making regarding comprehensive treatment strategies for this condition.

## Footnotes

1
http://www.gpower.hhu.de/
